# Voices of excellence: elite athletes' perspectives on support gaps, suggestions for improvement and their emergence

**DOI:** 10.3389/fspor.2025.1672311

**Published:** 2025-10-27

**Authors:** Alex Nico Griesinger, Peter Ehnold, Robert Zetzsche, Torsten Schlesinger

**Affiliations:** ^1^ Department Junior Elite Sport, Institute for Applied Training Science, Leipzig, Germany; ^2^ Institute of Human Movement Science and Health, Chemnitz University of Technology, Chemnitz, Germany; ^3^ Department of Sport & Management, IST-University of Applied Sciences Düsseldorf, Düsseldorf, Germany

**Keywords:** elite sport, junior elite sport, athlete support, holistic athlete development, biographical mapping

## Abstract

Elite athlete support is an area of elite sport development currently undergoing significant transformation, driven by athletes' demands for greater influence over the conditions under which they pursue their risky careers. Considering its potential to offer nations a competitive advantage, the field is continuously evolving and attracting growing strategic interest. The aim of the study is to examine elite athletes' unmet support needs, suggestions for improvement and their emergence to generate practically relevant insights and foster scientific knowledge. A qualitative design was applied, combining semi-structured interviews and biographical mapping with 23 German Olympic and Paralympic athletes from 12 sports. The data were analysed using content analysis and case-specific biographical-narrative reconstruction. Support needs and suggestions for improvement were individual and closely tied to athletes' life contexts, with most relating to training, dual career, financial and material support. Across support categories, the effectiveness of services depends on delivery quality as well as contextual factors such as information availability, communication and timing. In addition, athletes emphasise the need for interconnecting support measures and for earlier, more responsive support systems that adapt to changing circumstances. In this context, athletes highlight the central role of coaches in facilitating and ensuring support effectiveness. This study enriches holistic athlete support research by offering elite athlete-centred insights into the prevalence and emergence of support needs across careers. By integrating cross-case comparison with narrative depth, it delivers a rare blend of detailed and actionable insights from Olympic and Paralympic elite athletes that can inform more effective support systems in elite sport.

## 1 Introduction

The ever-increasing performance density in global elite sport requires the continuous realisation of success-promoting potential across all pillars of elite sport development for sporting nations, that want to be successful (1). The promotion and support of junior elite and elite athletes constitutes such a key success factor. Providing optimal conditions for athletes enables competitive advantages for nations, whereby maintaining these advantages requires constant adaptation of the support systems. Functioning support systems are a key feature of effective talent development environments (2). However, the mere provision of support services may be insufficient, as the effectiveness of such measures depends on the quality of the personnel involved, the nature of their relationships with athletes and the adoption of an integrated support approach, particularly given that athletes operate and develop within multiple environments simultaneously (3, 4). In addition, inadequate support can result in negative outcomes, such as inhibited athletic development or dropout (2, 5).

It can be observed that demands from athletes and their representatives to actively participate in the organisation of support systems have increased internationally (6). This involves the active participation of athletes in shaping policy that directly affect their support. Thereby, it touches more fundamental debates, such as overall athlete welfare systems and power relations between federations and athletes (7, 8). Developments in these key policy areas are evident at both international and national levels, with reforms progressing at different stages and shaped by diverse frameworks (7, 9, 10). This progress is driven not only by the shared objective of creating optimal conditions for the achievement of peak performance, but also by the growing recognition of the need to mitigate career-related risks faced by athletes. Athletes take on significant risks by focusing early temporally, thematically and socially on high-performance sport, without any guarantee of ultimately reaching or succeeding at the elite level (11). This biographical fixation on elite sport, coupled with a strong present-oriented mindset, often leads to the neglect of other life domains, such as formal education. The associated risks are frequently underestimated (12), with athletes accepting potential disadvantages for their post-sport careers, particularly concerning labour market opportunities (13).

Given the growing importance of athlete support and the increasing influence of athletes in policy development, it is pivotal to foster and expand knowledge that reflects their perspectives. To date, only few studies have examined the importance, prevalence and (potential) gaps in (junior) elite athlete support from the athletes' perspective (5, 14, 15). In response, this study follows calls for more athlete-centred research [e.g., (16, 17)] and analyses elite athletes' career trajectories, focusing on their (unmet) support needs, suggestions for improvement and how these needs develop over the course of their careers. To capture the complexity and variability of these experiences, a multi-perspective approach is applied, incorporating athletes from different career stages and from both Olympic and Paralympic sports. The aim of the study is to provide practical and actionable insights that enable athlete representatives and sports organisations to improve support systems, address existing gaps and implement more effective, needs-based solutions.

Against this background, the study addresses the following research questions: *(1) What unmet support needs and suggestions for improvement do elite athletes describe based on their career trajectories and lived experiences? (2) How do these needs develop?*

## 2 Literature review

International research on talent development environments, particularly shaped by studies following the Holistic Ecological Approach (18) or the Model of Effective Talent Identification and Development Procedures (19), has demonstrated that environmental factors (such as support measures) can contribute significantly to the successful and holistic development of athletes across countries (3). The effectiveness of such support services is contingent upon several factors, notably the extent to which the professionals involved are well-connected and engage in consistent communication with one another as well as with the athletes. This ensures the delivery of coherent messages, an up-to-date understanding of athletes' current situations (i.e., their perceptions and experiences) and the ability to coordinate support measures such as training, nutrition, recovery and psychological services accordingly (20, 21). The importance of this expert exchange increases particularly when athletes train across multiple environments, such as their club and the national team (4, 22). A reciprocal relationship of trust, which forms the foundation for individualised and high-quality support, constitutes a key prerequisite for its success (4).

In addition to the broader environmental perspective, studies specifically investigating the various areas and types of support are important to consider (23–25), of which athletes social support has gained great interest (i.e., support from friends and family). These studies are often conducted in the context of biographical life course research emphasizing the general importance of social support for holistic development and performance, especially with a view to overcoming difficult career phases (26–30). Also, many studies explore dual careers, assessing support measures within the context of different environments, such as elite sports schools (31, 32), university degree programmes or publicly funded institutions [e.g., Army, Federal Police; (33–38)]. Overall, support measures facilitating athletes' dual careers are nationally primed, yet critical and valued by most athletes across countries (24, 39–41). The value and characteristics of other support areas, such as medical care, psychological support, nutritional counselling and training and competition related support have been researched extensively (5, 21–23, 28, 42). Interestingly, despite the variety and quantity of support measures as well as their acknowledged importance (2, 29, 43), we find that athletes are aware of support programmes to varying degrees [i.e., psychology 30% to physiotherapy 79%; (5, 23)], which are also used to different extents by elite and junior elite athletes [i.e., psychology ∼22% to performance diagnostic ∼73%; (23, 25, 44, 45)]. Usage across almost all support areas has shown to increase with injury (42). In addition, (junior) elite athletes report deficits in their support schemes, especially with a view to education measures, finances, coaching, nutrition and medical care (15). Paralympic athletes emphasise the importance of medical care, physiotherapy and biomechanical performance diagnostics, with physical accessibility serving as a fundamental prerequisite (5). Athletes also report varying satisfaction levels with these support measures, which can change in significance throughout an athlete's career (14, 44).

Considering documented research gaps and partial dissatisfaction with existing support measures, as well as the potential negative consequences of inadequate support (e.g., prematurely dropouts), a stronger alignment with athletes' individual perspectives is needed. Standardised support systems run the risk of failing to adequately address the diversity and complexity of athletes' demands and needs. Recognizing that an athletes' path to elite sport is individual and shaped by the interaction between the athlete and his or her environment (46), a significant deficit is notable in research exploring the contextualisation and development of athletes' support needs. For example, the circumstances under which specific support becomes necessary throughout an athlete's career within temporal, social and factual contexts needs to be further explored. Such insights could prove highly valuable for sports systems, as they offer the potential to adapt standardised frameworks to better meet individual needs.

## 3 Theoretical framework

To address the research questions, we adopt the life course approach (47, 48), which assumes that an individual life course consists of a “sequence of activities across different life domains and institutionalised fields of action” [(49), p. 9], with the analytical focus placed on objective events and actions. Activities and events are not linear but interdependent and are influenced by previous actions and their outcomes as well as other contextual factors, resulting in a high degree of individuality, complexity and variability of life courses (49). Given its contextual stance, subjective attributions of meaning by the individuals are not inherently encompassed by the concept (48). To effectively analyse athletes' support needs and their emergence, it is essential to broaden the perspective and include the athletes' subjective and holistic viewpoints by incorporating biographical research methods (47, 50, 51). The added value of this combination lies in its ability to capture not only the connection and subsequent interaction of the athlete and his or her environment, as emphasised in ecological approaches (52), but also the temporal and biographical processes through which athletes interpret and respond to these influences. This enables a more nuanced understanding of how and why support needs and suggestions for improvement evolve over time, situated within individual life trajectories. Consequently, the theoretical considerations of life course research are empirically combined with biographical interviews (53) to analyse perceived support deficits, their emergence and the resulting recommendations for improvement.

Apart from the central supporting role of sport clubs, sports federations are responsible for the development and promotion of junior athletes in Germany (54). When athletes reach a certain age and performance level, regional sports federations become aware and support regional squad athletes, mainly through regular training sessions, training camps, material support and a yearly mandatory basic sports medical examination (55). Within these first years in junior elite sport, the aforementioned areas of training (science) and basic medical support attempt to foster the talents' sporting development while safeguarding their health. In addition, the admission to an elite sports school and attendance at a boarding school are support structures that enable advanced sporting development through more training, less travel times and customised schooling. Young athletes thus undergo continuous development across sporting and educational contexts, necessitating coordination among multiple stakeholders.

The transition from regional to national junior squad usually constitutes a critical phase for athletes, often accompanied by changes in their living, training and educational environment (56). Given many athletes struggle with these transitions, new support measures become available for athletes to better address associated challenges. With a view to performance development, the national federation provides regular training at the national training base, access to training camps and competitions. In addition, national squad athletes can access the services of the Olympic training centres (OSP) and other institutions (e.g., German Sports Aid Foundation or the Institute for Applied Training Science), which collectively offer the comprehensive range of support measures, including medical, psychological, nutritional and financial support as well as training or competition analyses and career counselling [see overview by (43)]. Moreover, state police forces, state fire brigades, the Federal Police, the German Armed Forces and German customs provide selected athletes with the opportunity to pursue a dual career. These roles offer a stable income and minimal work-related obligations during their active sporting careers, while ensuring long-term job security after retirement from sport. Partnerships with universities and other educational institutions support athletes through curricula tailored to the demands of elite sport (55).

On the one hand, these measures are designed to systematically develop the athletes' performance and exploit performance potential (i.e., using nutritional or psychological support). On the other hand, these measures also respond to the growing career risks associated with deeper integration into the elite sport system. An excessive degree of inclusion (hyperinclusion) in elite sports may limit individuals' opportunities for participation in other societal subsystems, most notably the educational system (11). Challenges such as limited private and social life or future educational disadvantages are addressed through educational and psychological counselling, financial support or employment with a federal institution. Interconnected support measures, such as close proximity between training and living environments or integrated nutritional support, are designed to enhance both performance development and athletes' overall life satisfaction. The objective is to organise the athletes' life in a way that enables and motivates them to achieve peak performance, while also maintaining satisfaction in other life domains and a positive outlook on their future. The achievement of this goal largely depends on the extent to which available support measures correspond to athletes' individual and evolving needs throughout the course of their careers.

## 4 Materials and methods

### 4.1 Research approach

This research adopts a constructivist perspective (57, 58). It assumes that a reality exists independently of individual consciousness. However, scientific inquiry can only approximate this reality, as individuals perceive and interpret the world through their own subjective and unique experiences. When a phenomenon is constructed and explained in similar ways by multiple individuals, this can be described as intersubjective knowledge. However, such shared understanding should not be equated with an objective, observer-independent truth. To explore the athletes' individual constructions and interpretations in depth and to effectively address the research questions, a qualitative research design was selected (59). This approach also enables the integration and critical reflection of existing theoretical perspectives throughout the research process. Rather than testing hypotheses deductively, the aim is to reconstruct and analyse participants' meaning-making within the context of relevant theoretical considerations (60). Moreover, the study adhered to consolidated criteria for reporting qualitative research [COREQ, see Supplementary Material; (61–63)].

### 4.2 Sampling

Purposive sampling was applied to select elite athletes for the qualitative interview study (64, 65). That is, we did not aim for statistical representativeness in the case selection, but defined certain theory-driven criteria based on the principle of contrast (61). To gain comprehensive insights into support deficits, suggestions for improvement and their development over time, athletes from a diverse range of Olympic and Paralympic winter and summer sports were recruited, encompassing both team and individual disciplines. Federations facilitated contact with athletes, aiming to include individuals with diverse career trajectories (e.g., early or late entry into the squad system, early or late developer; see additional criteria below). All contacted athletes participated in the interview (for more information, see Supplementary Material). These included national squad athletes in biathlon, canoe, diving, judo, nordic combined, para athletics, para canoe, para judo, para skiing, para swimming, rugby and table tennis. Furthermore, it was ensured that female and male athletes were included and that the athletes were at different stages in their sporting careers. The athletes were either actively competing, retired or had dropped out of elite sports, which we defined as having freely dropped out despite potential for elite performances has been acknowledged by their federation (66). A total sample of *n* = 23 athletes was generated, ranging from Olympic and Paralympic to national champions. In line with informed consent and to preserve anonymity, a basic description of the sample is provided in Table 1.

**Table 1:** Sample description.

Athlete (A)	Sport	Gender
A1	Biathlon	Female
A2	Biathlon	Male
A3	Canoe	Female
A4	Canoe	Female
A5	Canoe	Male
A6	Diving	Female
A7	Diving	Male
A8	Judo	Female
A9	Judo	Male
A10	Nordic Combined	Male
A11	Para Athletics	Female
A12	Para Athletics	Male
A13	Para Canoe	Female
A14	Para Canoe	Male
A15	Para Judo	Male
A16	Para Judo	Female
A17	Para Skiing	Male
A18	Para Swimming	Female
A19	Rugby	Male
A20	Rugby	Male
A21	Table Tennis	Male
A22	Table Tennis	Male
A23	Table Tennis	Male

### 4.3 Data collection

**Figure 1:**
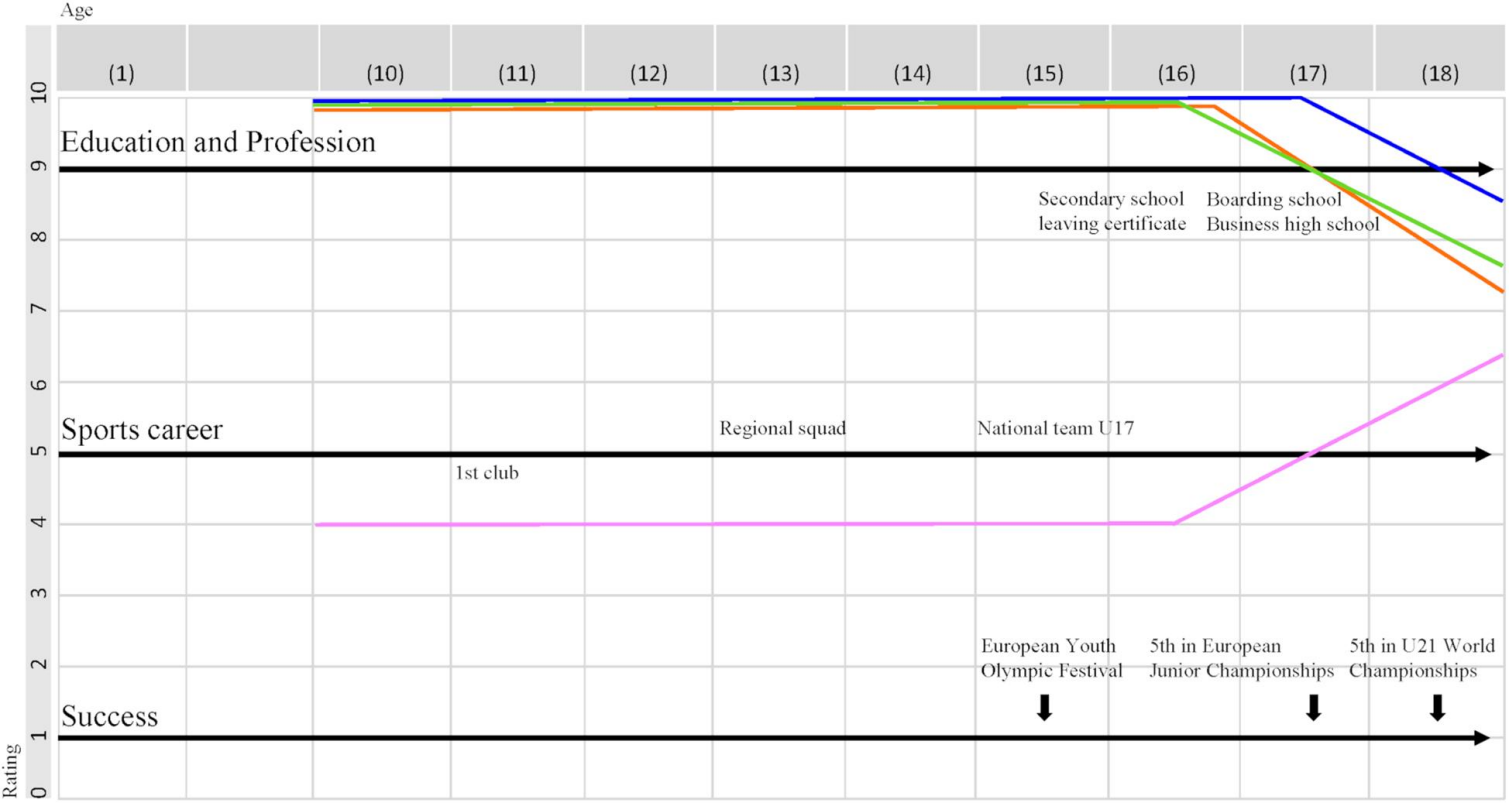
Career map. [pink] = sporting development (0 = stagnation, 10 = optimal development); [blue] = life satisfaction (0 = not satisfied at all, 10 = perfectly satisfied); [green] = health status (0 = not healthy at all, 10 = optimal health); [orange] = compatibility of education and sport (0 = not compatible at all, 10 = optimal compatibility).

The interview guideline was developed based on theoretical considerations and pre-tested to ensure clarity, coherence and practical feasibility (61). The athletes had ample space to provide narrative accounts and individual interpretations, allowing them to openly share their experiences about various support services, as well as about drawbacks and to offer suggestions for improvement (67). One challenge of data collection was the retrospective mapping of participants' support needs and recommendations for improvement. To address the methodological problems of memory gaps and biographical smoothing [i.e., unintended streamlining of narratives (68)], the interviews were supported with graphic visualisations based on biographical mapping [see Figure 1; (69–71)]. The biographical mapping instrument, which was developed based on similar studies (71) and adapted to the interview guideline, was also pre-tested during three pilot interviews (see Supplementary Material). Minor adjustments, e.g., linking the order of questions to related mapping tasks (e.g., biographical educational career in connection with compatibility of education and sport over time), resulted in smooth interview processes. Hence, a career map for each athlete was created from publicly available information prior to the interview (e.g., squad history, education, achievements) and supplemented with information during the interview. One interview was conducted in person and 22 via video conferencing (ZoomV5.9 software). The duration of the interviews varied between one hour eight minutes and three hours eleven minutes. Each participant gave a declaration of consent and granted permission for the interview to be digitally recorded and transcribed. To ensure anonymity, names, locations and personal details were omitted from the transcripts and replaced with placeholders. The aim of the de facto anonymisation is to change the data in such a way that “the person can only be re-identified with a completely disproportionate effort” [(72), p. 21].

### 4.4 Data analysis

The interview data were examined using a two-step qualitative design. First, content analysis was conducted, in which the reported support needs and suggestions for improvement were classified using a combination of deductive and inductive coding. Established support service categories (and respective subcategories) from the literature were used as the coding scheme for the deductive assignment of suggestions: nutritional science, training science, medicine, psychology, finances and material and dual career [see (43, 73, 74)]. That is, suggestions that addressed the quality or quantity of a specific support service (e.g., “more sport-specific nutritional counselling would have been beneficial”) were assigned to the corresponding predefined support category (support category “nutritional science”, subcategory “nutritional counselling and support”). In addition, **s**uggestions that were thematically linked to these support categories, but did not directly pertain to the measure itself (e.g., information availability, communication processes or timing), were assigned to the then inductively formed support-specific context category (e.g., “there was a no information on how to access psychological counselling”; support category “psychology”, subcategory “context-related proposals”), which resulted in an additional context-category per support category. Recommendations for improvement that could not be assigned to any of the support categories were grouped in an additional, inductive “miscellaneous” category, which ultimately included measures that only indirectly concern athlete support (e.g., increase in public attention). Second, through case-specific narrative reconstruction aided by biographical mapping, the athletes' identified support gaps and suggestions for improvement were examined in light of their subjective realities (e.g., perceived dual career compatibility) and in relation to various biographical tenets such as their squad status, educational status or significant life events (73). This approach provided valuable insights into the individual meaning-making processes underlying the athletes' suggestions. In accordance with this case-centred approach (75), the interpretative and subjective reasoning behind each suggestion was preserved and recorded in its entirety, while outlining the specific interplay of contributing factors and contexts case by case (76). Given the complexity of the data and spatial constraints, six cases (out of 23) are presented in detail. The individual cases under consideration (Athletes 3, 6, 8, 12, 17, 19) were chosen because they present excerpts from the career trajectories of various male and female Olympic and Paralympic athletes at different career stages and from different sports. They further differ in their suggested improvements across all areas of support, reflecting the diversity of perspectives and experiences. Moreover, the cases illustrate the individual nature of evolving needs and the underlying reasoning behind proposed improvements.

### 4.5 Quality criteria

As in quantitative research, qualitative research also applies specific criteria to ensure the quality of analysis (77). However, in contrast to quantitative approaches, no universally accepted set of quality criteria has yet been established within the field of qualitative research (61). Nevertheless, there is broad consensus regarding the importance of key principles such as traceability/consistency (reliability) and trustworthiness [validity; (61)]. In the present study, intersubjective traceability for external reviewers was ensured through a strong theoretical foundation, implemented via a structured interview guide and a systematic, rule-based content analysis (see data analysis and Supplementary Material). In addition, particular attention was paid to avoiding selective plausibility in the presentation of results (78). This was addressed, among other things, by supporting key findings with selected quotations from the interview data. The coding process, including definitions, anchor examples and coding rules, was independently reviewed and refined by two of the authors after approximately 30% of the interview material had been analysed (8 out of 23 cases). The feedback was subsequently discussed, used to refine the coding scheme and systematically applied to all interview material to ensure internal consistency and validate the coding framework [Stepwise Replication; (79)].

## 5 Results

### 5.1 Cross-case analyses—general suggestions for improvement

A total of 137 suggestions were extracted from the 23 interviews and systematically assigned to their corresponding support-related categories, with attribution to the respective athletes (see Table 2). Athletes who provided several suggestions within the same category were documented accordingly. Both male and female Olympic and Paralympic athletes submitted proposals across all categories (with no needs being assigned to seven subcategories), highlighting an accumulation of expressed needs in the areas of training science, dual careers and financial and material support.

**Table 2:** Suggestions for improvement—thematic categorisation adopted from Zetzsche, Griesinger (43); female athletes italicised.

Support categories	Subcategories	Athlete and sport	Number of proposals	
Nutritional science	Nutritional analyses	–		11
	Nutritional counselling and support	*A3* (Canoe), *A8* (Judo), A10 (Nordic Combined)	3	
	Sport-appropriate nutrition	–		
	Context-related proposals	*A3* (Canoe), *A6*, *A6* (Diving), *A8*, *A8*, A9 (Judo), A12, A12 (Para Athletics)	8	
Training science	Training-related support measures	*A1* (Biathlon), A7 (Diving), *A11*, A12 (Para Athletics), *A16* (Para Judo), *A18* (Para Swimming), A20 (Rugby)	7	28
	Training science support measures	*A6* (Diving), *A8* (Judo)	2	
	Diagnostics of motor abilities	–		
	Biomechanical performance diagnostics	–		
	Context-related proposals	*A1* (Biathlon), *A3*, *A3*, A5, A5 (Canoe), A7, A7 (Diving), A9, A9 (Judo), *A11*, *A11* (Para Athletics), A15, *A16*, *A16* (Para Judo), A17 (Para Skiing), A19, A19 (Rugby), A21, A22 (Table Tennis)	19	
Medicine	Medical diagnostics, treatments & similar	*A16*, *A16* (Para Judo), A17, A17 (Para Skiing)	4	10
	(Preventive/rehabilitative) therapeutic measures	*A4* (Canoe), A12 (Para Athletics), *A16* (Para Judo), A17 (Para Skiing)	4	
	Context-related suggestions	*A3* (Canoe), *A11* (Para Athletics)	2	
Psychology	Support for sporting matters	*A1* (Biathlon)	1	11
	Support for non-sporting matters	*A4* (Canoe)	1	
	Context-related proposals	*A6*, *A6* (Diving), *A8*, *A8*, *A8*, *A8*, *A8* (Judo), A12 (Para Athletics), A17 (Para Skiing)	9	
Finances and Material	(Monthly) monetary payments	*A4* (Canoe), *A6* (Diving), A12 (Para Athletics), *A13* (Para Canoe), A19 (Rugby), A23 (Table Tennis)	6	26
	Financial support for sporting activities	*A1*, A2, A2 (Biathlon), A7 (Diving), *A16* (Para Judo), A17 (Para Skiing), A22, A23 (Table Tennis)	8	
	Financial support for non-sporting activities	A15 (Para Judo), A19 (Rugby)	2	
	Context-related proposals	*A8*, *A8* (Judo), *A11* (Para Athletics), A14, A14 (Para Canoe), A15, *A16, A16, A16* (Para Judo), A17 (Para Skiing)	10	
Dual career	School-related support measures	A2, A2 (Biathlon), *A4, A4, A4* (Canoe), A14, A14 (Para Canoe), A22 (Table Tennis)	8	33
	Support measures in the transition from school to work	–		
	Support measures for vocational training	–		
	Support measures in higher education	*A6* (Diving), A10 (Nordic Combined), A12, A12 (Para Athletics)	4	
	Support via sports promotion groups/customs	A5 (Canoe), A9 (Judo)	2	
	Other education-related support measures	–		
	Context-related proposals	A2, A2, A2 (Biathlon), *A4* (Canoe), *A6* (Diving), *A8*, A9, A9 (Judo), A10 (Nordic Combined), *A13, A13* (Para Canoe), A15, *A16*, *A16* (Para Judo), *A18* (Para Swimming), A19, A19, A19 (Rugby), A22 (Table Tennis)	19	
Miscellaneous	Proposals not assignable to other categories	A2 (Biathlon), *A3* (Canoe), A7, A7 (Diving), *A8* (Judo), A14, A14 (Para Canoe), A15, A15, A15, *A16*, *A16*, *A16* (Para Judo), A17 (Para Skiing), *A18* (Para Swimming), A20, A20 (Rugby), A22 (Table Tennis)	18	18

In training science, several suggestions emphasised the need for more individualised training and context-specific needs, such as more and better coaching capacities and the upgrading of the coaching profession. Athletes also expressed the need for greater understanding of their elite sport-related responsibilities by educational institutions, ensuring their elite sport demands are accounted for. In the area of finance and material, athletes highlighted their need for financial security from the onset of their elite sports careers through retirement (e.g., pension entitlements during their active elite sports careers), as well as better financial resources to cover sport-related costs such as tournament fees, training equipment and transportation costs. It is noteworthy that suggestions for nutritional and psychological support came primarily from female athletes, who issued improvements to the systematic organisation of an initial contact, the quality of counselling for sport-related or sport-unrelated issues as well as adopting a holistic approach, e.g., sensitising coaches in this regard. In addition, suggestions for nutritional counselling were mostly mentioned by athletes in weight-sensitive individual sports. Paralympic athletes predominantly suggested improvements in medical support. This, for example, includes better access to physiotherapy, better classification as a specific para sport issue and closer coordination between health conditions and training plans, demonstrating potential for accommodating individual needs more effectively. The inductively developed category “Other” grouped recommendations that are not explicitly linked to direct support measures or their contexts (e.g., related communication). These recommendations are multifaceted, e.g., the call for investment in sports facilities, a support strategy that accounts for performance dips (without loss of support) as well as enhanced public recognition or more systematic approaches to talent search.

### 5.2 Individual case analyses

The following case analyses aim to contextualise athletes' experiences with support measures and corresponding suggestions for improvement. Moreover, they provide deeper insights into the individual circumstances that give rise to a specific need.

#### 5.2.1 Athlete 8

The successful judo career of athlete 8 has been marked by two particularly intense phases, which are contextualised in the following. The first phase occurred during her school years, particularly in the period leading up to her A-levels, characterised by an overwhelming dual burden.

“But by the end of my A-levels, I realised that I was through. I realised how difficult the double load had been because I was juggling judo, training camps and travelling. You just try to tick everything off, somehow getting your schoolwork over with”.

She did not have the option of extending her schooling years at high school, but she considered this to be useful support.

“I know that it’s not the same in many federal states. [In some] you can stretch it out to four years”.

The second phase of intense stress emerged after the Olympic Games, manifesting on a psychological level. Her relationship with a sports psychologist played a crucial role during this period. He supported her as she navigated recurring negative phases, which became particularly pronounced following the Games.

“Well, I’ve always had […] a bit of a winter depression […]. And that’s when I was confronted with what it’s like when you just don’t feel good. […]. But it wasn’t just one or two weeks, in fact, it turned into almost half a year. I just talked to him a lot”.

Besides, the psychologist helped her foster mental strength as an essential element of her sporting success.

“I would actually attribute all my successes to him. […] He really helped me use my mind as a weapon. He taught me how powerful the mind can be and what kind of strength it can actually have when it’s focused. That accounts for over 50 per cent of my performance when my mind is right there on the mat”.

Drawing from her experience in coping with difficult phases and maintaining her focus during competitions, she advocates for sports psychology support to be made accessible to all athletes, ensuring they have the opportunity to benefit from such counselling. More experienced athletes can play a mediating role in helping younger athletes find a psychologist they can trust and work with.

“So if I hadn’t taken the initiative myself, no coach would have told me, yes, go there now or anything like that. I believe sports psychologists are even more useful for younger aspiring athletes. But I think older athletes are better positioned to reach out to the younger ones than coaches, who simply assign a psychologist to you and say, alright, now work with them”.

She also believes that coaches should receive better training to understand and support athletes facing psychological difficulties.

“In my opinion, this aspect is, for whatever reason, not considered at all in the training of coaches. […] But it’s very difficult for coaches to understand. And yes, it’s also a learning process for them. So, if they’re better trained, then they can also recognise from the outside when someone is simply not doing well and (they can) talk to them, or create an alternative training plan for them”.

The importance of briefing coaches extends beyond psychology. In a sport where body weight plays an important role, this also applies to nutrition. Coaches also have a protective function in this regard:

“The physical aspect, that you somehow try to protect the younger ones from themselves. There are a lot of over-ambitious people who get carried away with things and hurt themselves, so a coach also has a protective function”.

Based on her personal experiences, the athlete emphasised the need for support in education, psychology and nutrition. Given the challenges she faced, intimate and high-quality counselling is particularly important to her, which she hopes can be passed on to younger athletes. The involvement of coaches in these processes is also important, which requires proper training and education as part of the coaching profession.

#### 5.2.2 Athlete 3

Athlete 3, an international medallist in canoeing, highlighted the significance of school-based support in fostering her athletic career. Specifically, she considered the one-on-one tuition she received during the final phase of her A-levels as particularly helpful and at the same time, valued it as a special privilege, given that the athlete was already competing internationally at the time.

“For the record, it must be stated quite clearly that we enjoyed a great privilege at school [in terms of support]”.

“In the last year, especially the last six months before my A-levels, I almost exclusively had private lessons because I was already part of the junior national team”.

In contrast, she identifies significant gaps in support for injury prevention and nutritional advice, especially from year 10 onwards due to the increasing amount of training.

“So if you then really commit to entering elite sport, the amount of training increases. I also think injury prevention would be much better if it were offered at that point or even at a younger age. In my experience and this is just about me personally, the nutritional counselling at the OSP was […] really poor. I simply didn’t have the financial means and didn’t want to burden my parents to find external help. I think it’s absolutely necessary to have high-quality staff. But if they’re not good, then you don’t need them”.

She also stated that her eating behaviour deviates from the norm, which is not uncommon in her sport. She suggests early awareness raising among coaches and qualified nutrition specialists to prevent eating disorders in young athletes:

“What is definitely an issue and is also a huge topic in our association at the moment, is the issue of disordered eating behaviour. I think it’s very important for coaches to be sensitised to this issue and are provided with guidelines on how to deal with it”.

“Especially when the individual is still at an impressionable young age, they don't yet have a solid personality. Yes, I believe there's a lot you can ruin or do wrong”.

In summary, this athlete's example highlights the importance of balanced and individualised support for junior elite athletes. While she largely viewed educational support as being positive, she's made bad experiences and identified gaps in nutritional counselling and injury prevention.

#### 5.2.3 Athlete 19

Athlete 19, a rugby player who had been attributed great potential by his federation, ultimately decided to drop out during his studies. For him, education has a very high priority and continuing in elite sport would only have been possible if it were compatible with his academic goals.

“Education has always been important to me and I was already aware that this switch in priorities would come at some point. That’s why I say that the only way I could have continued doing sport would have been if it had been compatible with education”.

It is important to note that the athlete had a strong commitment to the sport and would have pursued a dual career under certain conditions of support. However, he would have needed financial assistance, as the time demands of his sport made it impossible for him to take on a part-time job during his studies.

“Because sport takes up so much time, a mini-job during my studies would not have been possible and it would not have been feasible without financial support”.

Another important issue was the lack of opportunity to effectively combine studying and elite sport. There was no model that offered real compatibility between an academic and a sporting career.

“And the other issue is the compatibility with my studies and job, which wasn’t given for me. There wasn’t really any solution for me. The degree programme was also two years long. As I was thinking in terms of the Olympic cycle at the time, I knew that I would also be working afterwards and would have to be able to combine that with elite sport. And there wasn’t really an option for me, or at least I wasn’t shown one”.

Athlete 19 decided against continuing his rugby career due to the lack of compatibility between elite sport and studies, as well as insufficient financial support. Ultimately, the needs emerging from his expectations and aspirations beyond sport were not met by the support services offered.

#### 5.2.4 Athlete 17

Athlete 17, a Paralympic medallist in para skiing, describes that his career was significantly impacted by an injury, which severely limited him for several years. The injury was not only physically challenging, but also mentally stressful, as there were periods when he was unable to train for weeks and several surgeries were required.

“I was injured for a long time and it wasn’t clear if I would be able to continue in sport at all. Because I suffered a [the injury], which really cost me two and a half years of training and required a lot of extra effort from me, also a lot of mental effort, to keep going”.

His return to sport was a long process that required both discipline and the support of medical and physiotherapeutic measures.

“And during the injury phase, the physiotherapists and doctors were always available, always there and immediately looking for a solution”.

However, the experience made him more aware of the importance of close coordination between his medical history and individual training plan. The athlete believes that closer and sooner coordination between medical experts and coaches would have been necessary to prevent or mitigate such serious injuries.

“I think the only thing I would really, really change is the medical advice or tests in combination with the training plan and the performance. I’ve seen it with my [my injury]. Sure, something like that can always happen. It’s unfortunate. But I think that because things weren’t going so well for me at the time and we had simply trained a lot out of inexperience, we made some mistakes”.

Another difficulty Athlete 17 highlights is the taxation of financial support. The various funding pools and regulations create uncertainties and athletes need guidance.

“Because I went through three different funding modules last year alone, because they kept changing or other criteria applied, and then it was paid out from 5,000 different pots again, it was never clear. Because we receive all the money gross, or part of it is tax-free, anyway, but another part is not, and taxation is fully our responsibility. I’ve already heard from many people that they have no idea how to handle the taxes. It would be great to have a contact person who is familiar with the subject”.

The narrative accounts of Athlete 17 highlight his reasoning for his suggestions for improvement, which focus on dovetailing different areas of support as well as the provision of qualified financial counselling.

#### 5.2.5 Athlete 6

Athlete 6 enjoyed many years of international success, yet her career was also shaped by recurring phases where pressure became evident and support needs emerged. The first critical phase occurred in the pre-Olympic and Olympic year, when she failed to qualify for the Games, while simultaneously preparing for her final exams. Additionally, the one-to-one tuition she had received in previous years was no longer available.

“All of a sudden, I was expected to be at two places at once [competing in junior and senior events], but at the same time I was back in a regular class. That was a year when I realised just how privileged we had been over the previous three years [with one-to-one tuition]. Looking back, I’d say the planning wasn’t all that well thought through […]”.

Reflecting on the missed qualification, she describes having experienced a dip in motivation:

“The Olympic year was a year when I found it all a bit overwhelming. I’d been away a lot the year before and had built up hopes for the Olympics. Then I sort of fell into a motivational slump, because I felt I’d given everything and it still didn’t work out”.

At that time, psychological support services were already available through the federation, but she didn't use them. However, she later recognised that mental aspects were underestimated in her sport:

“I think in our sport, it’s still somewhat underestimated. And because of that, it’s not something that’s actively encouraged either. I did think about it at some point, but then it would’ve just been another appointment to squeeze into my week. So I think it’s something that just needs to be communicated a bit better”.

Another area where the athlete points to improvements is in the support areas of nutrition, as well as the interconnection of the menstrual cycle and training. She describes these issues as rarely being addressed with sufficient sensitivity or considered in training planning:

“Nutrition is naturally a central issue in our sport, given that it is aesthetic […] in nature. As a result, topics such as nutrition itself, dietary counselling, how to manage or reduce weight are more commonly taken up by the girls and women. I would definitely draw that distinction. Aside from that, there wasn’t really any structured support or counselling available in that regard”.

“In terms of training planning, [the menstrual cycle is] not at all [accounted for]. You’re first confronted with it in year five or six [in school], when you have to get into the water. Back then, we had a female coach, who was obviously more sensitive to it. But even then, it was more like, ‘talk to the older girls’. So it’s less about the coaches actively supporting you, it’s more pushed onto other female athletes”.

The athlete pleads for improvement in support and communication:

“It’s not something that’s ever really addressed. It all gets lumped into puberty, when your body changes, including nutrition and menstruation. What bothers me is how it’s talked about. Like you’re left to deal with it on your own and it’s not addressed with enough sensitivity. A typical example: “lose weight”. If you’re lucky, someone is assigned to support you. But often, the path you take doesn’t matter, as long as the result is right. I think these topics need to be handled more sensitively, with better support along the way”.

Athlete 6's journey illustrates how crucial individualised support is in high-performance sport, especially during the sensitive phases she encountered, such as educational transitions, sporting setbacks or pubescent development processes. She argues that simply offering support services is not enough; they must be actively communicated, well-coordinated and tailored to the athlete's life circumstances and needs.

#### 5.2.6 Athlete 12

Athlete 12 was active in football, when an experienced athlete introduced him to para athletics. After completing school, he undertook a voluntary social year at the Olympic Training Centre, lived in the affiliated residence and significantly increased his training frequency. He made daily use of the training facilities at the centre and was closely integrated into its support system. From his perspective, physiotherapy and osteopathy were key elements of support:

“I’ve actually been doing osteopathy since I was little. I know a good osteopath. I’ve recently started going again because I’ve had some back problems. Physiotherapy is directly at the OSP in […], so I could always make good use of it. It was right next door to where I was living”.

It was important to the athlete that these services were not offered as standardised treatments, but were specifically tailored to his physical needs. He sees this individual adaptation as crucial:

“Physiotherapy and osteopathy were adapted specifically for [my handicap]”.

A suggestion for improvement raised by the athlete relates to the quality of technical training. In hindsight, he criticised the fact that the training camps he attended as part of the junior national squad included little specific instruction in technique. Instead, general training dominated the sessions:

“I felt that when I was in the junior squad and went to those camps, it was mostly general training. The problem was that no one there really knew about [technique], so I didn’t learn much”.

The athlete emphasises that specialised coaches who can address technical requirements in a targeted manner would significantly improve both the appeal and effectiveness of training. Moreover, he identifies issues around nutritional support in the residential setup. Although he lives and trains near the boarding facility, he is not allowed to take part in evening meals, which are restricted to boarding school residents:

“At our residence, there’s always dinner, but only the boarding school kids are allowed to eat there, not those in the residence. Cooking takes a lot of time. For example, when we’re at training camps and don’t have to deal with meals and can focus completely on sport, I notice how much better everything goes. You don’t have to worry about anything and can concentrate fully on training. That’s ideal. But we don’t have that here”.

Athlete 12's experience highlights how effective institutional integration and individualised support can be in building an athletic career. His statements also reveal that, despite well-structured support measures, not all needs are fully met and performance potential could have been leveraged to a greater extent.

## 6 Discussion

### 6.1 Discussion of the results

The advancement of support systems for junior elite and elite athletes constitutes a key objective pursued by ambitious sporting nations, driven by its potential as a competitive advantage and by athletes' demands for greater involvement in shaping related policies (1, 80). Our results indicate that athletes face unmet support needs across all areas of support outlined in the literature: nutritional science, training science, medicine, psychology, finances and material and dual career (43). This applies to both male and female Olympic and Paralympic athletes, allowing for an inclusive categorisation and consideration of support measures, except for accessibility constituting a prerequisite for many Para-athletes to access such measures (5). Notably, the majority of needs identified in our study align closely with those reported in other studies (15), particularly in the areas of training science, finances and material and dual careers (14, 44).

Similar to other studies (44, 81), communicated needs in training science in this study focus on frequent, high-quality and individualised training, in particular targeting individual contextual circumstances, that prevent or negatively influence this “desired training”. For example, athletes have highlighted the need for greater coaching capacities to better address individual training demands, including gender-specific considerations such as those relevant to female athletes. While para-specific needs about training did not emerge prominently in our study (i.e., para-specific knowledge of coaches), they have been identified in other research (5). Moreover, the athletes emphasised the importance of higher qualifications for coaches, as well as improving the reputation of the coaching profession in general, to make it more attractive and increase the number of highly qualified coaches. Needs targeting coaches' capacities extend beyond training science; athletes expressed unmet demands for coaches to play an important role as communicators or facilitators of other support services (e.g., psychological and nutritional support) and thus underline the significance of inter-individual aspects of the coach-athlete relationship as well as the well-researched importance of regular communication between coaches and experts (2, 30, 82, 83). The underlying factors related to coaches' capacities and qualifications arise from a perceived scarcity of resources, as do the unmet needs regarding finances and materials.

Many athletes emphasise the need for financial security (for retirement), as well as coverage of costs associated with their sport (such as equipment and transport). This need is particularly relevant for junior athletes to alleviate the financial burden on their parents. There is reason to assume that financial relief, such as reduced burden on athletes' families and prospects of future financial security, may mitigate the high-risk career trajectories associated with an elite sport career (44, 80). However, these needs may vary across different sports and athletes, for example depending on the costs of their sport (81).

Dual career needs are individual and, as demonstrated in this and other studies, can pose significant risks for athlete dropout, if not adequately addressed (14, 37). The demands are aimed at creating dual career opportunities and improving the balance between educational and sporting careers. These demands are inherently heterogeneous, ranging from the desire for increased academic support to specific requests, such as the rescheduling of exams during police training. From the athletes' perspective, long-term, personalised support coupled with a systematic effort to enhance understanding of elite sports within educational institutions is desirable (81).

Likewise, the delivery of high-quality, individualised “services' is a decisive criterion for effective consultation-based nutritional and psychological support measures. Beyond the relevant service itself, the value of the support measure is determined (or obstructed) by general contextual conditions, i.e., how and when contact is established, the ease and efficiency of communication and the level of support provided. These contextual factors vary widely and differ in significance depending on the athlete's personal circumstances. Immediate and intensive counselling becomes essential if these needs arise from a severe negative mental state experienced by the athlete (25). Although athletes' needs are individual, the provision of information about when they “are allowed” to access certain support measures and whom they should turn to for such support should follow a systematic approach. In this study, the process was described as fragmented and insufficient, reflecting similar findings reported in other studies (5, 23). Thus, several athletes expressed a need for improved communication and for earlier initial contact in these areas of support (29, 84), which were used and emphasised by female athletes, in particular (15, 25).

The necessity for health services frequently originates from injuries and illnesses, as noted by Emrich, Güllich (42). However, within our sample, such necessities were infrequently cited. When they were mentioned, it was predominantly by Paralympic athletes. Given their individual circumstances, Paralympic athletes naturally have more extensive medical needs, which primarily focus on increased access to physiotherapy and more comprehensive, customised preventive medical screenings (5, 85). Overall, the low number of responses in this category indicates that, with some exceptions, the medical care provided to most athletes in our study did not leave room for improvement. This is noteworthy given the importance of medical support, particularly for developing junior athletes and in view of the Covid-19 pandemic, which had a profound impact on the lives of Olympic and, in particular, Paralympic athletes. Not only health-related, but with a view to athletes' psychosocial wellbeing, financial security and training and competition opportunities (85–87).

The examination of the development of unmet needs and recommendations for enhancement indicates that these needs emerge across all areas of support, stemming from the unique circumstances and lived experiences of the athletes. This is unsurprising, as these needs are the outcome of an interplay of multiple factors (29, 48). The personal significance that athletes attribute to their career paths, their experiences within those paths and their outlook on the future significantly shape how they perceive and evaluate potential support measures. That is, whereas one athlete may regard psychological counselling as a peripheral option, another may rely on it as both an emotional anchor and a structural necessity for performing or managing the transition out of elite sport. This individual and varying significance attributed to specific support measures poses major challenges for support systems and the way in which these challenges are addressed appears to be a key determinant of their overall effectiveness.

Despite the outlined individuality of needs (development), overarching factors such as gender or sport specific characteristics seem to exist and may allow certain need patterns to be anticipated, such as financial support in materially intensive sports or nutritional counselling in weight sensitive disciplines. As indicated in the theoretical considerations, squad transitions and the life changes inherently linked to them, appear to be significant with regard to the necessity of support measures. Such tendencies were evident and described in the present study, but have been reported even more prominent in other research (81). Although the results are based on the assessments of German athletes, we consider these conclusions to be internationally relevant. While athletes, their paths and the resources available to them might differ considerably, the sports they practise, the sporting goals they pursue and the competition environments they share a certain point in time, are likely to induce similar needs to some extent.

### 6.2 Practical implications

Based on the results, the following recommendations for practical guidance can be formulated: (1) Athletes need to be systematically informed about the support services they are eligible to access. In this context, a mandatory initial appointment or proactive outreach appears to be an effective measure for reducing potential barriers and leveraging holistic development, regardless of whether the service is ultimately utilised. (2) Athletes' holistic needs should be systematically addressed when creating support programmes. Given the dynamic nature of the athletes' life courses, their needs and the meaning they assign to them, close monitoring and frequent reporting cycles focused on capturing these are recommended. Ideally, an “athlete manager” systematically monitors their individual circumstances and responds to the athlete's specific needs within the scope of available possibilities. (3) Athletes' individual needs should be addressed with flexibility (rather than through formalised and standardised support systems) in terms of access to support measures. From the athletes' perspective, some support measures are only offered when it is already too late (e.g., nutritional advice, psychological support, career counselling). It is evident that such substantial policy changes can only be implemented over the long term and through political discourse involving a range of stakeholders. These include, for example, a loosening of the link between national squad status and eligibility for support or greater flexibility in the use of federations' funds for athlete support, which in Germany is partly constrained by prescribed subsidy purposes, yet currently under discussion (10). In this context, decentralised solutions at the club or regional level, or within the private sector, which are less bound to structural conditions, appear more practicable in the short term. In addressing this challenge, in addition to meeting the communicated needs, opportunities for holistic development could be leveraged. (4) Since many athletes express needs in relation to training and given its overall significance, elite sports systems should prioritise the provision of optimal, individualised training conditions for athletes, with a focus on delivering high-quality coaching resources, also with a view to coaches' central role in leveraging other support services.

### 6.3 Limitations and future research

In addition to the insights discussed, the methods employed in the study also present limitations. (1) The qualitative approach and the complexity of athletes' careers makes it difficult to analyse a large number of cases across different sports. Hence, the results only allow for a deduction of assumptions that must be further validated. Specifically, it can be assumed that other career paths could offer additional valuable insights into gaps in support mechanisms (problem of theoretical saturation). Since federations facilitated access to the athletes, highly critical perspectives may have been underrepresented due to selection or general social desirability bias. Moreover, recall bias, which we addressed using biographical mapping, cannot be entirely ruled out. Based on these considerations and the results of our study, a further and more comprehensive investigation into athletes' needs is recommended. This could involve larger sample sizes (for life course research), more differentiated data structures and analytical methods to examine and empirically support the recommendations derived. (2) Considering the national context is essential for ensuring the practical relevance of the results. Nonetheless, both the findings and the methodological design employed are applicable in an international context. From this perspective and given certain need patterns might be systematically addressed, an analysis of cross-national differences in athletes' support evaluations, along with an inquiry into whether these differences stem from particular system characteristics, for example the maturation of mechanisms facilitating athlete involvement in decision-making, would be of interest. Considering the limited number of available studies and the current development towards greater athlete co-determination rights, more (international) studies that focus on athletes' needs in the context of national elite sport systems and that inclusively address Paralympic athletes are necessary.

## Data Availability

The datasets presented in this article are not readily available because data protection requirements permit disclosure only under specific conditions. Requests to access the datasets should be directed to Alex Griesinger, griesinger@iat.uni-leipzig.de.
