# Supplementary material for: Voices of excellence: elite athletes' perspectives on support gaps, suggestions for improvement and their emergence
Source: Front Sports Act Living. 2025 Oct 27;7:1672311. doi: 10.3389/fspor.2025.1672311 (PMC12597968; doi:10.3389/fspor.2025.1672311)
Supplement: Supplementary file 1 [file Table1.pdf]

Supplementary Material

Table 1

Consolidated criteria for reporting qualitative research (see 1).

No.	Item	Guide questions/ description	Answer
Domain 1: Research team and reflexivity			
Personal Characteristics			
1	Interviewer	Which authors conducted the interviews?	Robert Zetzsche, Alex Griesinger
2	Credentials	What were the researcher's credentials? e.g., PhD, MD	Robert Zetzsche [Diploma Sociologist, PhD Student], Alex Griesinger [Master of Science Sport Management, PhD Student]
3	Occupation	What was their occupation at the time of the study?	Both were researchers at the Institute for Applied Training Science [IAT].
4	Gender	Was the researcher male or female?	Male (both)
5	Experience and training	What experience or training did the researcher have?	Both researchers had prior experience in designing and conducting interview studies.
Relationship with participants			
6	Relationship established	Was a relationship established prior to study commencement?	There was no relationship with any participant before the interviews.
7	Participant knowledge of the interviewer	What did the participants know about the researcher? e.g., personal goals, reasons for doing the research	The researchers and the aim of the study were introduced as part of the participant briefing prior to the interview.
8	Interviewer characteristics	What characteristics were reported about the interviewer/facilitator? e.g., Bias, assumptions, reasons and interests in the research topic	Before the athletes were contacted by the researchers, they were contacted by their federations and given a rough outline of the study. It is unclear whether and to what extent the characteristics of the researchers were discussed.
Domain 2: study design			
Theoretical framework			

9	Methodological orientation and Theory	What methodological orientation was stated to underpin the study? e.g., grounded theory, phenomenology, content analysis	The study employed a qualitative approach, combining content analysis with case-specific biographical-narrative reconstruction.
Participant selection			
10	Sampling	How were participants selected? e.g., purposive, convenience, consecutive, snowball	Purposive sampling (see *Sampling*)
11	Method of approach	How were participants approached? e.g., face-to-face, telephone, mail, email	Participants were approached by email and telephone (contact information and permission to contact organised by the sports federations).
12	Sample size	How many participants were in the study?	23
13	Non-participation	How many people refused to participate or dropped out? Reasons?	None
Setting			
14	Setting of data collection	Where was the data collected? e.g., home, clinic, workplace	Online (for 22 participants) and face-to-face (one participant).
15	Presence of non-participants	Was anyone else present besides the participants and researchers?	Yes, the interviewer was accompanied by additional researchers (see *Acknowledgements*, colleagues at the IAT), who complemented the athletes' biographical mapping based on the information given during the interview.
16	Description of sample	What are the important characteristics of the sample? e.g., demographic data, date	Participants had to be elite athletes and given the aim of capturing wide-ranging perspectives, various theory-driven criteria were defined (see *Sampling*).
Data collection			
17	Interview guide	Were questions, prompts, guides provided by the authors? Was it pilot tested?	The interview guideline was developed based on theoretical considerations and pilot tested with three other athletes (face-to-face and online).
18	Repeat interviews	Were repeat interviews carried out? If yes, how many?	No
19	Audio/visual recording	Did the research use audio or visual recording to collect the data?	Yes, the online interviews were audio and video recorded with the athletes' consent using ZoomV5.9.

20	Field notes	Were field notes made during and/or after the interview or focus group?	Yes, a biographical map drafted prior to the interview was completed during the interview (see *Data collection*).
21	Duration	What was the duration of the interviews or focus group?	The interviews lasted between 68 and 191 minutes.
22	Data saturation	Was data saturation discussed?	Yes, data saturation was discussed by the authors. The researchers do not assume saturation of the data, as only a limited number of athletes from certain sports were available for the interviews.
23	Transcripts returned	Were transcripts returned to participants for comment and/or correction?	The biographical maps were sent to and commented by the athletes (see 28).
Domain 3: analysis and findings			
Data analysis			
24	Number of data coders	How many data coders coded the data?	Two researchers coded the data (see *Data analysis*).
25	Description of the coding tree	Did authors provide a description of the coding tree?	Yes, the coding tree is outlined in Table 2.
26	Derivation of themes	Were themes identified in advance or derived from the data?	Support categories were deducted from the literature and inductively advanced by respective context-categories and an additional other-category (see *Data analysis*).
27	Software	What software, if applicable, was used to manage the data?	MAXQDA 2020
28	Participant checking	Did participants provide feedback on the findings?	Yes, the final draft of the biographical career map was to be confirmed by the athletes.
Reporting			
29	Quotations presented	Were participant quotations presented to illustrate the themes / findings? Was each quotation identified? e.g., participant number	Yes, participant quotations accompanied by an athlete code are used to illustrate the findings (see *Individual case analyses*).
30	Data and findings consistent	Was there consistency between the data presented and the findings?	Yes (see *Cross-case analyses* and *Individual case analyses*).
31	Clarity of major themes	Were major themes clearly presented in the findings?	Yes, Table 2 provides an overview about the general results.
32	Clarity of minor themes	Is there a description of diverse cases or discussion of minor themes?	Yes, see *Cross-case analyses* and *Individual case analyses* for in-depth insights into diverse cases and

findings beyond the results shown in Table 2.

1. Tong A, Sainsbury P, Craig J. Consolidated Criteria for Reporting Qualitative Research (Coreq): A 32-Item Checklist for Interviews and Focus Groups. *International Journal for Quality in Health Care* (2007) 19(6):349-57.
